# Biomass and Wastes for Bioenergy: Thermochemical Conversion and Biotechnologies

**DOI:** 10.1155/2018/9638380

**Published:** 2018-11-01

**Authors:** Ningbo Gao, Lei Zhang, Chunfei Wu

**Affiliations:** ^1^School of Energy and Power Engineering, Xi'an Jiaotong University, Xi'an 710049, China; ^2^School of Environmental Science & Technology, Dalian University of Technology, Dalian 116024, China; ^3^School of Chemistry and Chemical Engineering, Queen's University Belfast, Belfast BT7 1NN, UK

In response to the global environmental pollution and energy resource issues, biomass and wastes have obtained a widespread attention to be used for fuel and energy production. Now it is evident that wastes that could cause risks to the environment could be converted into useful energy via some realistic technologies. Using biomass and wastes as an energy resource not only solves environmental pollution problems, but also reduces the dependency on fossil fuels. Thermochemical and biological conversion of biomass and wastes are the two most important bioenergy conversion methods.

In order to pursue the latest developments of bioenergy and keep the global academic communities up to date to the current advances in the Biomass and Wastes for Bioenergy, the applications of biomass in thermochemical conversion and biotechnologies have been discussed in 8 high-quality papers published in this special issue. The brief introduction for these works is listed as follows.

The paper titled “Energy Analysis of a Complementary Heating System Combining Solar Energy and Coal for a Rural Residential Building in Northwest China” by X. Zhen et al. designed a work on energy efficiency of the system and the determination of thermal efficiency of a coal stove by using a prototype model. In this study, multiple linear regression was adopted to present the dual function of multiple factors on the daily heat-collecting capacity of the solar water heater. Their results showed that the orientation and the shade of solar water heaters had profound influences on heat-collection capacity compared to the reference solar water heater. Additionally, the allocation of the radiation of solar energy projecting into the collecting area of the solar water heater was only effectively utilized by 28%. Moreover, the results also showed that the main factors that led to the high heat loss were the mismatch between the working temperature of the radiator, collecting temperature of the solar water heater and the location of storage tanks.

The paper titled “Experimental Study on Productivity Performance of Household Combined Thermal Power and Biogas System in Northwest China” by J. Kang et al. developed a method to create a heat, electricity, and biogas cogeneration system with low-temperature solar thermal collectors, photovoltaic solar power generators, and solar-powered thermostatic biogas digesters. The method was experimentally studied via two buildings in a farming village in northwestern China. Even though the ambient temperature reached down to −25°C, the temperature of the biogas digester was maintained at 27°C±2 for thermostatic fermentation. After optimization, the energy-saving rate was improved from 66.2% to 85.5%. This installation reduced CO_2_ emissions by approximately 27.03 t, and the static payback period was 3.1 yr. The results indicated that the system was highly economical, energy efficient, and beneficial for the environment.

The paper titled “Neural Network Prediction of Corn Stover Saccharification Based on Its Structural Features” by L. Gao et al. developed a neural networks model, which was demonstrated for the prediction of the corn stover saccharification based on the features without enzymatic hydrolysis. By using this method, the predicted value of corn stover digestibility was very similar to the actual determined value of corn stover digestibility. A fast approach for bioenergy crops selection could be offered by the neural network model. Thus, the neural network model is cost-effective and time-saving and it will have a good application for corn stover storage and evaluation.

The paper titled “Ni-Ru/CeO_2_ Catalytic Hydrothermal Upgrading of Water-insoluble Biocrude from Algae Hydrothermal Liquefaction” by D. Xu et al. used hydrothermal liquefaction to convert wet algae into water-insoluble biocrude and other coproducts. By using Ni-Ru/CeO_2_+H_2_, the yield, HHV (higher heating value) and the best elemental compositions for quality of water-insoluble biocrude (B_2_) were increased. The result indicated that the Ni-Ru/CeO_2_+H_2_ technology was able to transform high-molecular-weight compounds into low-molecular-weight compounds and led B_2_ to contain a series of abundant aliphatic saturated hydrocarbons such as pentadecane, tridecane, tetradecane, heptadecane, and dodecane. The results also showed that Ni-Ru/CeO_2_+H_2_ led to the largest fraction and the best light of light biocrude in B_2_.

The paper titled “Second-Generation Bioethanol from Coconut Husk” by M. Bolivar-Telleria et al. discussed the methods that have been used to produce bioethanol from coconut husk and suggested ways to improve different aspects of the process. It was observed that the use of enzymes to perform hydrolysis was a good alternative. In this review, it has been confirmed that alkaline pretreatment is the best choice for delignification potential. Biorefining of this material for the production of ethanol and other molecules with greater added value would enable economically developing countries to create new jobs and boost income.

The paper titled “The Effect of Digested Manure on Biogas Productivity and Microstructure Evolution of Corn Stalks in Anaerobic Cofermentation” written by Z. Lv et al. explored the effect of mixing corn stalk with cow dung at five different fermentation stages on the further cofermentation process. The straw microstructure evolution was investigated by the SEM and XRD methods to identify the optimal conditions for the straw biodegradation process enhancement. The authors proved that the mesophilic codigestion of corn stalk with cow dung improved the biogas production, enhanced the degradation efficiency of organic matter, and reduced the anaerobic fermentation cycle. The results of this study were quite instrumental to the further optimization of the corn stalk anaerobic digestion by inoculation with digested manure for lignocellulose degradation enhancement and biogas productivity improvement.

The paper titled “The Effects of Different Oxytetracycline and Copper Treatments on the Performance of Anaerobic Digesters and the Dynamics of Bacterial Communities” by Y. Zhang et al. evaluated the performance of anaerobic digesters and discussed the dynamics of bacterial communities under the different treatments of oxytetracycline and copper during the anaerobic digestion of cow manure. Methane production and pH value were measured and analyzed to reflect the performance of anaerobic digestion. PCR-DGGE method was used to discuss the dynamics of bacterial communities. It was found that the bacterial communities had significant differences under the different treatments of oxytetracycline and copper.

The paper titled “The Migration and Transformation of Heavy Metals in Sewage Sludge during Hydrothermal Carbonization Combined with Combustion” by M. Liu et al. investigated the migration and transformation behaviors of heavy metals (Cr, Mn, Ni, Cu, Zn, As, Cd, and Pb) during the hydrothermal carbonization of sewage sludge. In this work, the authors considered the effects of HTC reaction temperatures and residence times on the distribution and chemical speciation transformation behaviors of HMs in the HTC of sewage sludge. The results indicated that most of the HMs accumulated in the Solid residue (SR) during HTC and combustion process. The authors also concluded that the HTC process promoted the immobilization of HMs in the combustion process.

